# Restoration of Electric Footshock-Induced Immunosuppression in Mice by *Gynostemma pentaphyllum *Components

**DOI:** 10.3390/molecules17077695

**Published:** 2012-06-25

**Authors:** Sun-A Im, Hyun Sook Choi, Soon Ok Choi, Ki-Hyang Kim, Seungjeong Lee, Bang Yeon Hwang, Myung Koo Lee, Chong Kil Lee

**Affiliations:** College of Pharmacy, Chungbuk National University, Cheongju 361-763, Korea; Email: sunaim@chungbuk.ac.kr (S.-A.I.); 6151494@hanmail.net (H.S.C.); pa960001@cnuh.co.kr (S.O.C.); yes3343@hanmail.net (K.-H.K.); chdang@chol.com (S.L.); byhwang@chungbuk.ac.kr (B.Y.H.); myklee@chungbuk.ac.kr (M.K.L.)

**Keywords:** *Gynostemma pentaphyllum*, electric footshock, stress, immunomodulation, cytotoxic T lymphocytes

## Abstract

The immunomodulatory effects of the ethanol extract of *Gynostemma pentaphyllum *(GP-EX) were examined in electric footshock (EFS)-stressed mice. The mice were orally administered various doses of GP-EX for 7 days before exposure to EFS (duration: 3 min, interval: 10 s, intensity: 2 mA) once a day from day 8 for 14 days with continuous daily feeding of GP-EX. Oral administration of GP-EX to mice prevented EFS stress-induced immunosuppression as determined by the lymphoid organ (thymus and spleen) weight and cellularity. In addition, oral administration of GP-EX restored EFS-suppressed functional properties of mature lymphocytes in terms of concanavalin A-induced proliferation of splenocytes and lipopolysaccharide-induced cytokine production (TNF-α, IL-1β). Furthermore, we found that mice that were orally administered with GP-EX generated much more potent ovalbumin-specific cytotoxic T lymphocyte responses upon intravenous ovalbumin injection compared to the untreated controls. These results demonstrate that oral administration of the ethanol extract of *Gynostemma pentaphyllum* could increase host defense in immunocompromised situations such as stress-induced immunosuppression.

## 1. Introduction

*Gynostemma pentaphyllum* is a perennial creeping herb of the genus *Gynostemma*. The plant belongs to the Cucurbitaceae family, which includes cucumbers, gourds and melons [1]. There are 21 species of *Gynostemma* and the *G*. *pentaphyllum*, the most prevalent species, is dispersed throughout Asia including China, Korea and Japan [1,2]. *Gynostemma pentaphyllum* is usually used as an herbal tea in Southern Asia. In folk medicine, *Gynostemma pentaphyllum *has been used to treat a variety of diseases such as diabetes, depression, anxiety, fatigue, hyperlipidemia, immunity, oxidative stress and tumors [2,3,4,5,6,7,8,9,10]. 

The dammarane (triterpenoid) saponins isolated from *Gynostemma pentaphyllum* are believed to be the active components responsible for its various biological activities and reported clinical effects. Saponins isolated from *Gynostemma pentaphyllum* are also well-known as gypenosides. So far, about 169 dammarane-type saponins, *i.e*., gypenosides, have been isolated from the plant [11,12,13]. These gypenosides number approximately five or six times more than the ginsenosides, the major bioactive components of *Panax ginseng*. In addition, nine gypenosides have been identified: the protopanaxadiol-type ginsenosides Rb1, Rb3, Rc, Rd, Rg3, F2, malonyl-Rb1 and malonyl-Rd, and one saponin, ginsenoside Rf, a protopanaxatriol-type ginsenoside [2,3,12]. Thus, *Gynostemma pentaphyllum* has more diverse dammarane-type saponins than any other plant.

The stress response occurs via very complex and multifaceted mechanisms involving a series of physiological, behavioral, metabolic, and immunological reactions [14,15,16,17,18]. In mammals, the integrated response to stress includes a number of neuroendocrine molecules of both the hypothalamic-pituitary-adrenal axis and the sympathetic nervous system, such as catecholamines, adrenocorticotropin and glucocorticoids [19,20,21]. 

Immune cells such as leukocytes and antigen-presenting cells carry stress hormone receptors produced by the adrenal and pituitary glands. Stress conditions are associated with increased plasma corticosterone levels because corticosterone is the end product of the hypothalamic-pituitary-adrenal axis. For that reason, these hormones can modulate the immune cell functions, the proliferation of splenocytes in response to antigenic and mitogenic stimuli, and the production of proinflammatory cytokines and nitric oxide [21,22]. It has also been reported that corticosterone is responsible for many quantitative and qualitative changes in immune functions [23,24,25,26]. The most well-known effect of stress on the immune system is the atrophy of immune organs and the suppression of immune cell functions [27,28,29,30,31,32]. Electric footshock is the most commonly used stressor in animal studies to model the stress-induced changes in humans [33,34,35,36,37].

GP-EX has been shown to restore dexamethasone-induced immunosuppression in mice [38]. GP-EX also has been shown to have anti-stress effects in electric footshock stress (EFS)-induced mice [33]. In the present study, the immunomodulatory effects of GP-EX were examined in EFS-stressed mice as well as in normal mice. Our results show that the oral administration of GP-EX restores EFS-induced immunosuppression.

## 2. Results

### 2.1. GP-EX Prevents EFS Stress-Induced Atrophy of Lymphoid Organs

To examine the effects of GP-EX on the EFS stress-induced immunosuppression, mice were orally administered with GP-EX (10, 30 and 50 mg/kg/day) for 7 days and then subjected to a daily session of EFS stress from day 8 (duration: 3 min, interval: 10 s, intensity: 2 mA) for 14 days with continuous daily feeding of GP-EX.

**Figure 1:**
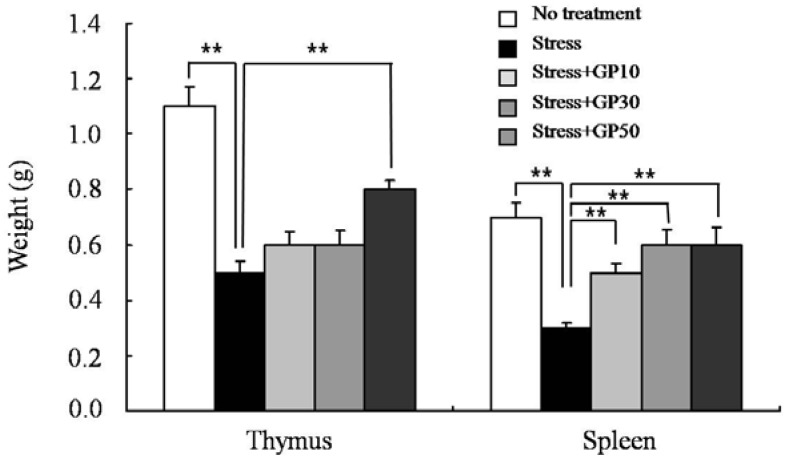
GP-EX prevents EFS stress-induced atrophy of lymphoid organs. Mice were pretreated with GP-EX (10, 30 and 50 mg/kg/day, p.o.) at 10:00 for 7 days and then subjected to a daily session of EFS stress at 14:00 from day 8 (duration: 3 min, interval: 10 s, intensity: 2 mA) for 14 days with continuous daily feeding of GP-EX at 10:00. The mean lymphoid organ (thymus and spleen) weights were measured after sacrificing the mice on day 22. The results are presented as means ± SEM of three experiments (n = 10). ** *p* < 0.01, compared to control levels.

After sacrificing the mice on day 22 following the initiation of GP-EX feeding the mean lymphoid organ (thymus and spleen) weights were measured. As shown in Figure 1, EFS stress significantly decreased the average weights of thymus and spleen compared with that of the unstressed group. However, oral administration of GP-EX for 21 days significantly attenuated the EFS stress-induced atrophy of lymphoid organs compared with the untreated group. The preventive effect of GP-EX on the stress-induced atrophy of lymphoid organs was prominent in the spleen (Figure 1).

### 2.2. GP-EX Restores EFS Stress-Induced Disturbances in Lymphocyte Cellularity

**Figure 2:**
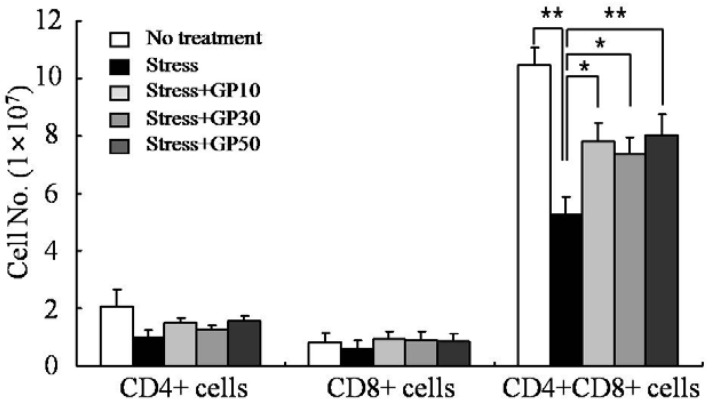
GP-EX restores EFS stress-induced disturbance in thymus cellularity. Mice were stressed with EFS and treated with GP-EX as described in Figure 1. Then the cellularity of the thymus was measured by flow cytometry after sacrificing the mice on day 22. The cells were collected, washed, and then used for immunophenotypic analysis. * *p* < 0.05, ** *p* < 0.01 compared to control levels.

The cellularity of CD4+ T cells, CD8+ T cells and CD4+CD8+ T cells in the thymus was investigated by flow cytometry. As shown in Figure 2, EFS stress decreased CD4+CD8+ T cells to approximately 48% compared to that in unstressed mice. Oral administration of GP-EX (10, 30, 50 mg/kg/day) for 21 days significantly restored the cellularity of CD4+CD8+ T cells in EFS-stressed mice. Although there was no statistical difference, oral administration of GP-EX also increased the cellularity of CD4+ T cells and CD8+ T cells in EFS-stressed mice. 

**Figure 3:**
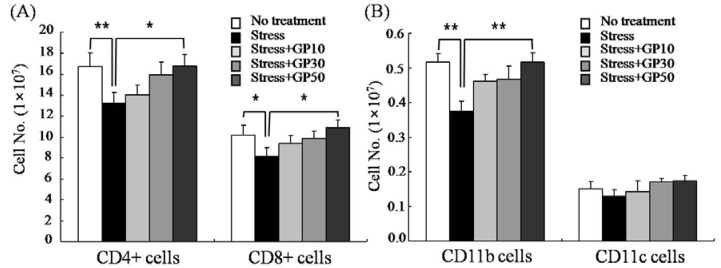
GP-EX restores EFS stress-induced disturbances in spleen cellularity. Mice were stressed with EFS and treated with GP-EX as described in Figure 1. Then the cellularity of the spleen was measured by flow cytometry after sacrificing the mice on day 22. The cells were collected, washed, and then used for immunophenotypic analysis. * *p* < 0.05, ** *p* < 0.01 compared to control levels.

The cellularity of CD4+ T cells, CD8+ T cells, CD11b+ cells and CD11c+ cells in the spleen was also examined by flow cytometry. There were significant decreases (20~25% decrease) in the cellularity of CD4+ T cells, CD8+ T cells and CD11b+ cells in the spleen of EFS-stressed mice (Figure 3). Oral administration of GP-EX (50 mg/kg/day) for 21 days significantly restored the EFS-induced decreases in the cellularity of CD4+ T cells, CD8+ T cells and CD11b+ cells. Although not statistically significant, oral administration of GP-EX appeared to restore the cellularity of CD11c+ cells in the spleen of EFS-stressed mice.

### 2.3. GP-EX Restores EFS Stress-Induced Decreases in LPS-Induced Cytokine Production

**Figure 4:**
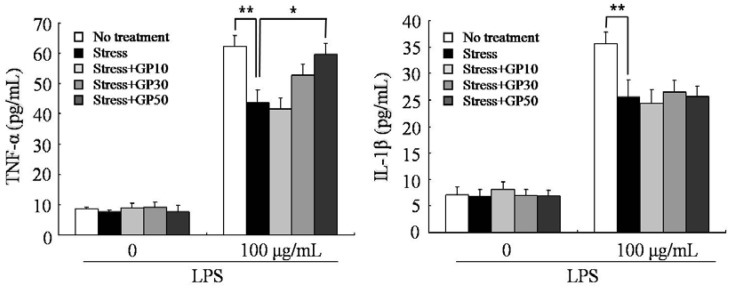
GP-EX restores EFS stress-induced decrease in LPS-induced cytokine production. Mice were stressed with EFS and treated with GP-EX as described in Figure 1. Peripheral blood was obtained from the vein after sacrificing the mice on day 22, diluted five-fold with cell culture medium and incubated in the presence of LPS (100 μg/mL) for 48 h. The levels of cytokine in the blood culture supernatant were measured by ELISA. Values are means ± SD. * *p* < 0.05, ** *p* < 0.01 compared to control levels.

In this experiment, the effects of GP-EX on the EFS stress-induced suppression of cytokine production by peripheral blood lymphocytes were also examined. Using heparin as an anticoagulant, peripheral blood was obtained from the vein and diluted five-fold with medium. The whole blood was incubated in the presence of LPS (100 μg/mL) for 48 h and the release of TNF-α and IL-1β was measured by ELISA. EFS stress decreased the production of TNF-α and IL-1β by 25~30% compared with that in unstressed mice (Figure 4). Oral administration of GP-EX (50 mg/kg/day) for 21 days significantly restored the stress-induced suppression of TNF-α production by LPS stimulation. Oral administration did not exert restorative effects on the decrease of IL-1 β production by EFS stress.

### 2.4. GP-EX Restores EFS Stress-Induced Deceases in Splenocyte Proliferation

**Figure 5:**
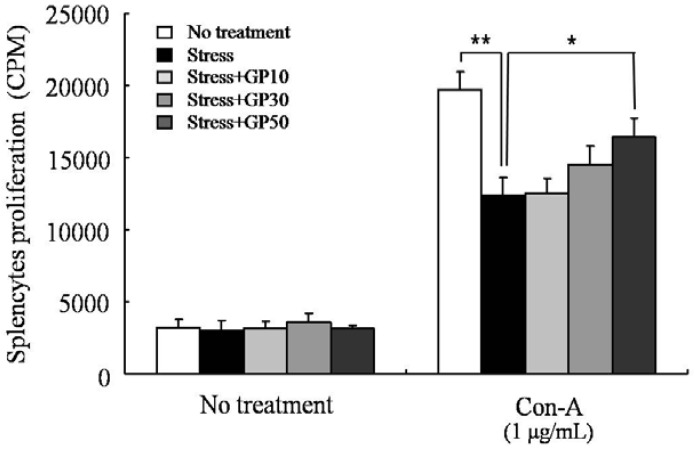
GP-EX restores EFS stress-induced deceases in splenocyte proliferation. Mice were stressed with EFS and treated with GP-EX as described in Figure 1. Total spleen cells (1 × 10^6^/well) from the mice were cultured in the presence of Con A (1 μg/mL) for 3 days. DNA synthesis of splenocytes was measured by ^3^[H]-thymidine incorporation for the final 18 h of the 3-day culture period. Values are means ± SD. * *p* < 0.05, ** *p* < 0.01 compared to control levels.

The effects of oral administration of GP-EX on the proliferation of splenocytes were also examined in EFS-stressed mice. As shown in Figure 5, Con A-induced proliferation of splenocytes was decreased by approximately 38% in EFS-stressed mice compared with that in unstressed mice. This EFS stress effect was restored by oral administration of high concentration of GP-EX (50 mg/kg/day). However, low concentration of GP-EX (10, 30 mg/kg/day) did not have significant restorative effects. 

### 2.5. GP-EX Restores EFS Stress-Induced Deceases in OVA-Specific CTL Activity

**Figure 6:**
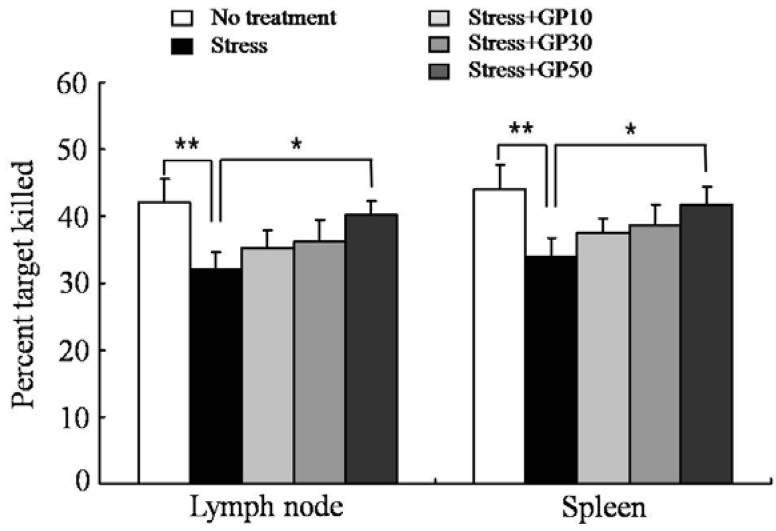
GP-EX restores EFS stress-induced deceases in OVA-specific CTLs. Mice were stressed with EFS and treated with GP-EX as described in Figure 1. OVA (100 μg) was injected intravenously on day 15. To analyze OVA-specific cytotoxicity, the cells from the spleens of naive syngeneic mice were pulsed with OVA_257–264_ peptide and labeled with a high concentration of CFSE (CFSE^high^). To control for Ag specificity, unpulsed syngeneic cells were labeled with a low concentration of CFSE (CFSE^low^). A 1:1 mixture of each target cell population was injected (i.v.) into recipient mice on day 21 and specific cytotoxicity was determined 18 h later. Values are means ± SD. * *p* < 0.05, ** *p* < 0.01 compared to control levels.

The effects of oral GP-EX administration on the generation of antigen-specific CTLs were examined in EFS-stressed mice. Mice were pretreated with GP-EX (10, 30 and 50 mg/kg/day) for 7 days and then subjected to a session of EF stress once a day from day 8 (duration: 3 min, interval: 10 s, intensity: 2 mA) for 14 days with continuous daily feeding of GP-EX. Each mouse was immunized (i.v.) with soluble OVA (100 mg/mouse) on day 15, and OVA-specific CTL activity was measured 7 days later using an *in vivo* CTL assay. EFS stress markedly suppressed the induction of OVA-specific CTL activity. As shown in Figure 6, oral administration of GP-EX (50mg/kg/day) for 21 days significantly restored the OVA-specific CTL generation in EFS-stressed mice.

## 3. Discussion

The major finding of the present study is that the oral administration of GP-EX restores EFS-induced immunosuppression. We examined the immunomodulatory activities of GP-EX in EFS-stressed mice. Oral administration of GP-EX in mice restored EFS stress-induced immunosuppression as determined by the lymphoid organ (thymus and spleen) weight, cellularity, Con A-induced proliferation of splenocytes, LPS-induced cytokine production (TNF-α, IL-1β) and induction of OVA-specific CTL activity. 

EFS-stress significantly decreased the average weights of thymus and spleen compared with that of the unstressed group. In this model, we showed that oral administration of GP-EX significantly attenuated the EFS stress-induced atrophy of lymphoid organs compared with the untreated group. Examination of the cellularity of the thymus showed that the major cell type that was diminished in the thymus of the EFS-stressed mice was CD4+CD8+ immature thymocytes, and the oral administration of GP-EX significantly restored the EFS stress-induced death of CD4+CD8+ immature thymocytes. The majority of T cells in the thymys are CD4+CD8+ immature T cells [39], which are sensitive to steroid hormones [40]. Thus, it is tempting to speculate that EFS-stress induced the production of steroid hormones, and the steroid hormones induced the apoptotic death of immature thymocytes. For single positive mature T cells, EFS-stress and also the oral administration of GP-EX did not result in significantly changes in the cellularity in the thymus. However, as shown in Figure 2, although there was no statistical difference, EFS-stress decreased the cellularity of mature T cells in the thymus, and the oral administration of GP-EX increased the cellularity of CD4+ T cells and CD8+ T cells in EFS-stressed mice. It is noteworthy that thymus is a central lymphoid organ generating mature T cells, and thus minor differences in the thymic cellularity of mature T cells could result in significant differences in the peripheral cellularity. The effects of GP-EX on the functional properties of mature lymphocytes were also examined in EFS-stressed mice. We showed that oral administration of GP-EX restored EFS-suppressed lymphocyte functions including mitogen-induced proliferation and the generation of antigen-specific cytotoxic T lymphocyte.

Based on many studies of the relationships between various stressors and immune parameters, different stressors can have different effects on immune parameters depending on the nature, intensity and time delay between the stressor and the immune parameter studied [41,42]. Stressor stimuli exert profound changes on cellular and humoral immune function in animals [42]. For example, exposure of rodents to EFS or restraint has consistently been shown to depress whole blood proliferative responses to lymphocyte mitogens such as Con A and phytohemagglutinin (PHA). The nonspecific proliferative response of rat splenocytes is decreased after a single session of EFS, with significant suppression occurring after 3–5 shock sessions [43,44]. Exposure to a 3-h session of tail shock also decreases spleen cell proliferation and IL-2 production [45]. Acute or repeated inescapable EFS exposure 5–7 days after cholera toxin immunization suppresses non-specific spleen cell proliferation, while augmenting the proliferative response to specific antigen [46]. Exposure to acute and long-term EFS of various intensities results in an intensity-dependent decrease in nitric oxide production but an increase in the IL-1β production by alveolar macrophages [47]. 

Plant extracts have been used since ancient times in traditional medicine for various diseases. Recently, interest in phytochemicals with anti-stress activity has been increasing. Oral administration of *Pueraria tuberosa* tuber extract and *Withania somnifera *rhizome extract showed protective effects against chronic EFS stress-induced physiological, neurobehavioral and neuropathological alterations. Chronic EFS stress significantly altered the behavioral patterns, decreased the sexual urge and activities, damaged the gastric mucosal layers, enhanced the plasma corticosterone levels and increased the weights of the adrenal glands and spleen. *Pueraria tuberosa* tuber extract and *Withania somnifera* rhizome extract showed significant anxiolytic activity, protected the gastric mucosa, lowered plasma corticosterone levels and restored the hypertrophy of adrenals and spleen [48]. Chronic restraint stress exerted cognitive dysfunction, altered behavioral parameters, increased leukocyte counts, superoxide dismutase, lipid peroxidation, glucose and corticosterone levels, and decreased catalase and glutathione reductase activities. The ethyl acetate-soluble fraction of *Morus alba* restored all these chronic restraint stress-induced disturbances [49]. The flavonoids, quercetin and kaempferol, isolated from the ethanol extract of *Ayurvedic rasayana*, increased the capacity to tolerate restraint-induced acute (3 days) and chronic (7 days) stress and swimming-induced stress [50]. The anti-stress effects of the leaves of *Alchornea cordifolia* were based on their ability to increase tolerance to the duration of immobility and the forced swim endurance test [51]. The extract of *Ptychopetalum olacoides* (Marapuama) prevented the unpredictable chronic mild stress-induced anxiety and hyperglycemia. In addition, the extract of *Ptychopetalum olacoides* significantly increased the time to hypoxia-induced convulsion [52]. Oral administration of *Ginkgo biloba* and *Panax ginseng* extracts decreased the acute restraint stress-induced adrenal hypertrophy, hyperglycemia, creatine kinase, and circulating corticosterone levels. *Ginkgo biloba* and *Panax ginseng* extracts have potent adaptogenic activities that are mediated by regulation of adrenal and pituitary adrenocorticotropic hormone secretion by cortical cells [53]. The water extract of *Sarcandra glabra* improved restraint stress-induced immunosuppression via its antioxidant function. Oral administration of *Sarcandra glabra* extract restored the stress-induced decreases of the number of lymphocytes and the balance of CD4(+) T/CD8(+) T and NK cell activity in mice spleen. The water extract of *Sarcandra glabra* also significantly decreased the level of lipid peroxidation and increased the oxygen radical absorbance capacity in splenocytes [54].

In this study, we investigated the immunomodulatory effects of the ethanol extract of *Gynostemma pentaphyllum *(GP-EX) in EFS-stressed mice. GP-EX has been reported to have anti-stress activity in mice. Oral administration of GP-EX significantly restored the decreased body weight, grip strength, endurance and catecholamine levels in EFS-stressed mice compared with untreated EFS-stressed mice [33]. GP-EX has also been reported to have immunomodulatory activity in mice. GP-EX treatment prevented dexamethasone-induced immunosuppression in mice as determined by the mitogen-induced proliferation of splenocytes and the LPS-induced cytokine production (TNF-α, IL-1β) in the whole blood culture. GP-EX has also been shown to increase the antitumor host defense in mice implanted with sarcoma-180 tumor cells [38].

GP-EX contains dammarane-type saponins. The major components of GP-EX have been identified as gypenoside derivatives, which have anti-inflammatory activity [55], protective effects against glutamate-induced neurotoxicity [56], and protective effects against hydrogen peroxide-induced oxidative stress [57]. Therefore it is tempting to speculate that gypenoside derivatives in GP-EX are the active components exerting immunomodulatory activity in EFS-stressed mice.

## 4. Experimental

### 4.1. Preparation and Treatment of GP-EX

*Gynostemma pentaphyllum* was obtained from Geochang (Gyungnam, Korea) and a voucher specimen of the herbal leaves of *Gynostemma pentaphyllum* was deposited at the herbarium of the College of Pharmacy, Chungbuk National University (Cheongju, Korea). The air-dried leaves of *Gynostemma pentaphyllum* were extracted with ethanol (70%, v/v, 18 L/10 kg, 24 h at room temperature) and the ethanol extracts were evaporated to dryness under reduced pressure and temperature (GP-EX, 1.05 g; yield, 10.5%, w/w) [3]. The dry GP-EX was suspended in water for the experiments. GP-EX (10, 30 and 50 mg/kg) was administered to mice orally (p.o.) once a day for 21 days. 

### 4.2. Animals

Six-week-old C57BL/6 male mice were purchased from Orient BioCo. (Seoul, Korea) and were acclimated for 1 week. The mice were housed five per cage in a conventional system at a room temperature of 20~22 °C, humidity of 50~65%, 12 h:12 h light/dark cycle with 150~300 lux and had free access to commercial pellet food and purified water. All experimental procedures were approved by the Animal Care Committee of Chungbuk National University.

### 4.3. Cell Culture

Cells were cultured in Dulbecco’s modified Eagle’s medium (Hyclone Laboratories Inc., Logan, UT, USA) supplemented with 10% heat-inactivated fetal bovine serum (Hyclone), 100 U/mL penicillin and 100 μg/mL streptomycin (Hyclone), and 50 μM 2-mercaptoethanol (Sigma-Aldrich, Inc., St. Louis, MO, USA) at 37 °C in 5% CO_2_ atmosphere. 

### 4.4. EFS Stressor

Mice (C57BL/6, male, 18–20 g) were pretreated with GP-EX (10, 30 and 50 mg/kg/day, p.o.) or vehicle (0.9% saline, p.o.) at 10:00 for 7 days and then subjected to a daily session of EFS stress at 14:00 from day 8 for 14 days with continuous daily feeding of GP-EX at 10:00. Mice subjected to inescapable scrambled EFSs (duration: 3 min, interval: 10 s, intensity: 2 mA) were placed in one of two compartments of a Plexiglas shock box (24 cm long, 24 cm wide, and 32 cm high; Shock generator, Seil Electric Co., Daejeon, Korea) with a grid floor. The control groups of animals remained in their home cages throughout the experiments. 

### 4.5. Flow Cytometry

Cells were stained with monoclonal antibodies recognizing murine cell surface markers as described previously [58], and flow cytometric analysis was performed on a FACSCanto II (BD Sciences, San Jose, CA, USA). The monoclonal antibodies, anti-CD4 (clone GK1.5), anti-CD8 (clone 53–6.7), anti-CD11b (clone M1.70) and anti-CD11c (N418), and isotype-matched control antibodies were purchased from BD Biosciences. Dead cells were gated out by their low forward angle light scatter intensity. In most analyses, 10,000 cells were scored.

### 4.6. Growth Stimulatory Activity

Total spleen cells were prepared from spleen of C57BL/6 mouse and single-cell suspensions were isolated using a 70-μm cell strainer (BD Falcon). The single cell suspension was washed with PBS, and then red blood cells were lysed by treatment with ACK lysis buffer (0.15 M NH_4_Cl, 1.0 mM KHCO_3_, 0.1 mM EDTA) for 3 min. The spleen cells were washed, and then cultured in 96-well microtiter plates (2 × 10^5^/well) in a volume of 200 μL per well in the presence or absence of Con A (1 μg/mL) for 3 days. DNA synthesis was measured by [^3^H]-thymidine (Du Pont, 0.5 μCi/well) incorporation for the final 18 h of the culture period.

### 4.7. Peripheral Blood Cytokine Production

Using heparin as an anticoagulant, peripheral blood was obtained from the vein and diluted five-fold with Dulbecco’s modified Eagle's medium (Hyclone) containing 2.5 IU heparin (Sigma-Aldrich), 100 IU penicillin/100 mg streptomycin (Hyclone) per mL. After addition of the bacterial stimulus LPS (Sigma-Aldrich), mouse whole blood was incubated in polypropylene vials (BD Falcon) in the presence of 5% CO_2_ at 37 °C for 2 days. After incubation, the blood cells were pelleted by centrifugation (400 ×g, 10 min) and the cell-free supernatants were stored at −70 °C for cytokine determinations. The amounts of IL-1β and TNF-α in the culture supernatants were measured using commercially available ELISA kits (BD Biosciences). 

### 4.7. *In Vivo* CTL Assay

Mice were immunized (i.v.) with soluble OVA (100 mg/mouse), and OVA-specific CTL activity was measured 7 days later. Target cells for *in vivo* evaluation of cytotoxic activity were prepared as described previously [58]. Briefly, naive C57BL/6 spleen cells were either pulsed with 10^−6^ M OVA[257–264] peptide for 1 h at 37 °C and then labeled with a high concentration of CFSE (5 µM), or just labeled with a low concentration of CFSE (1 µM). An equal number of cells from each population were mixed together and injected (i.v.) into immunized recipient mice (1 × 10^7^ cells/mouse). Specific *in vivo* cytotoxicity was determined by flow cytometry for the lymph node and spleen cells isolated from the recipient mice 18 h after i.v. injection. The ratio of the percentages of uncoated OVA[257–264]-coated (CFSE^low^/CFSE^high^) was calculated to obtain a numerical value for cytotoxicity. 

### 4.8. Statistical Analysis

Data were expressed as mean ± SD. The statistical significance of the difference between the control group and treatment group was assessed by one-way ANOVA followed by a Tukey’s test.

## 5. Conclusions

The ethanol extract of *Gynostemma pentaphyllum* could increase host defense in immunocompromised situations such as stress-induced immunosuppression. However, further studies are needed to investigate the active components and biological mechanisms involved in the restorative activity of GP-EX on the immunosuppression induced by EFS.
